# Complete Chloroplast Genome Sequence of *Dendrobium nobile* from Northeastern India

**DOI:** 10.1128/genomeA.01088-16

**Published:** 2016-10-06

**Authors:** Ruchishree Konhar, Devendra Kumar Biswal, Manish Debnath, Sriram Parameswaran, Durai Sundar, Pramod Tandon

**Affiliations:** a Bioinformatics Centre, North-Eastern Hill University (NEHU), Shillong, Meghalaya, India; b Genotypic Technology (P) Ltd., Bangalore, Karnataka, India; c Department of Biochemical Engineering and Biotechnology, Indian Institute of Technology Delhi, New Delhi, India; d Department of Botany, North-Eastern Hill University (NEHU), Shillong, Meghalaya, India

## Abstract

The orchid species *Dendrobium nobile* belonging to the family *Orchidaceae* and genus *Dendrobium* (a vast genus that encompasses nearly 1,200 species) has an herbal medicinal history of about 2000 years in east and south Asian countries. Here, we report the complete chloroplast genome sequence of *D. nobile* from northeastern India for the first time.

## GENOME ANNOUNCEMENT

The genus *Dendrobium* contains many highly prized medicinal plants. *Dendrobium nobile* Lindl. is one such endangered medicinal plant listed in the Convention on International Trade in Endangered Species of Wild Fauna and Flora (CITES) Appendix II and therefore calls for urgent attention from conservationists and evolutionary biologists alike toward devising control measures for its preservation and propagation. Per traditional medicinal practices, different parts of *D. nobile* are used as an antidepressant, aphrodisiac, analgesic, antipyretic, and for curing gastrointestinal diseases (1–3). Of late, several studies have reported *D. nobile* phenanthrenes with anticancerous properties (4).

Complete chloroplast genomes of 912 land plants have been reported in National Center for Biotechnology Information (NCBI) Organelle Genome Resources (http://www.ncbi.nlm.nih.gov/genome/browse/?report=5). We have determined the chloroplast genome sequence of *D. nobile* and report it for the first time from northeastern India. The complete chloroplast genome of *D. nobile* was determined from a whole-genome project initiative of the same species by paired-end and mate-pair data from Illumina HighSeq with 150 × 2 and Illumina NextSeq500 with 75 × 2, respectively. The *D. nobile* plant specimen was collected from National Research Centre for Orchids, Sikkim of northeastern India. Voucher specimen was deposited in the Department of Botany, North-Eastern Hill University, Shillong and Botanical Survey of India. Fresh young leaves were taken from *D. nobile* orchids grown in a greenhouse. High molecular weight DNA was extracted using a modified CTAB buffer. For next-generation sequencing (NGS) library preparation, both paired-end and mate-pair libraries were prepared and quantified using Qubit and validated for quality by running an aliquot on D1000 ScreenTape (Agilent). Libraries were amplified for nine to 11 cycles per Nextflex protocol, and quantified and sequenced on Illumina NextSeq500. Perl scripts developed in-house were used for preprocessing of data reads, adapter clipping, and quality filtering. The raw reads were trimmed with error probability <0.001 and assembled using CLC Genomic Workbench 7.7.1. Protein-coding tRNA and rRNA genes were annotated using CGAP (5) and DOGMA (6). The boundaries of each annotated gene were manually determined by comparison with orthologous genes from other orchid chloroplast genomes. The circular genome maps were drawn using OGDRAW (7). The genome was annotated with a total of 132 genes, which includes eight rRNA genes, 38 tRNA genes, 79 protein-coding genes, and seven pseudo-genes. The chloroplast genome has a quadripartite structure with two inverted repeat regions of 26,285 bp, one large single copy (LSC) of 84,944 bp, and a small single copy (SSC) region of 14,504 bp. Twelve genes were found to have introns (rps16, atpF, rpoc1, ycf3, rps12 [2], clpP, petB, rpl2 [2], ndhB [2]), and 20 genes were found to be duplicating in the inverted repeats.

### Accession number(s).

The complete chloroplast genome sequence of *D. nobile* with all annotated genes has been submitted to GenBank under the accession number KX377961.
